# The pathogenesis and therapeutic strategies of heat stroke-induced myocardial injury

**DOI:** 10.3389/fphar.2023.1286556

**Published:** 2024-01-08

**Authors:** Rui Xia, Meng Sun, Yuling Li, Jing Yin, Huan Liu, Jun Yang, Jing Liu, Yanyu He, Bing Wu, Guixiang Yang, Jianhua Li

**Affiliations:** ^1^ Department of Critical Care Medicine, Chongqing University Jiangjin Hospital, Chongqing, China; ^2^ Department of Anesthesiology, Union Hospital, Tongji Medical College, Huazhong University of Science and Technology, Wuhan, China; ^3^ Emergency Department, The First Affiliated Hospital of Dalian Medical University, Dalian, China; ^4^ Nanjing Jinling Hospital, Affiliated Hospital of Medical School, Nanjing University, Nanjing, China

**Keywords:** heat stroke, myocardial injury, pathogenesis, therapeutic strategy, inflammation

## Abstract

Heat stroke (HS) is a febrile illness characterized by an elevation in the core body temperature to over 40°C, accompanied by central nervous system impairment and subsequent multi-organ dysfunction syndrome. In recent years, the mortality rate from HS has been increasing as ambient temperatures continue to rise each year. The cardiovascular system plays an important role in the pathogenesis process of HS, as it functions as one of the key system for thermoregulation and its stability is associated with the severity of HS. Systemic inflammatory response and endothelial cell damage constitute pivotal attributes of HS, other factors such as ferroptosis, disturbances in myocardial metabolism and heat shock protein dysregulation are also involved in the damage to myocardial tissue in HS. In this review, a comprehensively detailed description of the pathogenesis of HS-induced myocardial injury is provided. The current treatment strategies and the promising therapeutic targets for HS are also discussed.

## 1 Introduction

Heat stroke (HS) is an illness characterized by a rapid rise of core temperature over 40°C with the complication of systemic inflammatory responses and central nervous system dysfunction (Bouchama and Knochel, 2002; Leon and Helwig, 2010; Peiris et al., 2017). In recent years, heat-related deaths have increased significantly due to anthropogenic climate change (Toutant et al., 2011). Frequency of severe heat waves is threatening human health worldwide and poses huge challenges to public health, attracting widespread attention in various research fields (M. Zheng et al., 2020). HS can be divided into classic heat stroke (CHS) and exertional heat stroke (EHS) depending on the involvement of skeletal muscle contraction (Bouchama et al., 2022). CHS often occurs in older people having pre-existing illnesses, while EHS typically occurs in healthy younger individuals during strenuous exercise in hot environments (Peiris et al., 2017; Bouchama et al., 2022). HS, regardless of the type, is associated with extensive multi-organ tissue damage as a result of the interaction of cytotoxic, inflammatory, and clotting reactions (Piver et al., 1999). The heart, being a vulnerable organ in heat injury (Low et al., 2011; Lou et al., 2019; Ko et al., 2020), is susceptible to arrhythmia, function failure and focal myocardial necrosis (Argaud et al., 2007; Desai et al., 2023).

**FIGURE 1:**
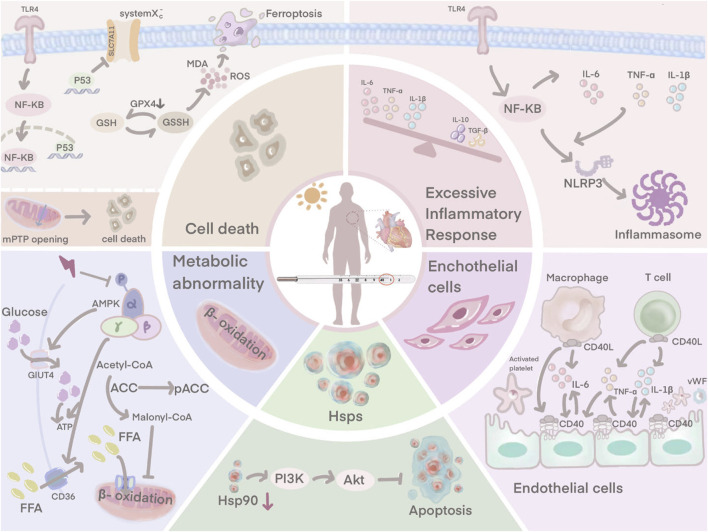
HS-induced myocardial injury is not only associated with an excessive inflammatory response, endothelial cell damage. Ferroptosis, downregulation of HSP90 expression and disturbances in cardiomyocyte metabolism are also involved.

Abnormalities in temperature regulation, cardiovascular function and tissue perfusion are among the factors involved in multiple organ dysfunction syndrome (Low et al., 2011; Cramer et al., 2022). In an effort to dissipate heat, the body increases blood flow to the skin, redistributes blood and eventually develops hypotension and perfusion disorders (S. H. Chen et al., 2006). Thus, the regulation of the cardiovascular system plays a key role in the pathogenesis of HS. Elucidation of the mechanism of HS-induced myocardial injury can help in establishing the treatment to improve circulatory function and reduce mortality rates of HS. However, the pathogenesis of HS is still to be known and prevention strategies of myocardial injury during HS is lacking. This article provides a systematic review to further the understanding of HS-induced myocardial injury and to provide a reference for future research (Figure 1).

## 2 Heat stroke and myocardial injury

Under normal conditions, a 0.3°C increase in core temperature triggers a cardiovascular regulatory response to protect the body from heat damage. This regulation increases heat dissipation by speeding up the heart rate, enhancing cardiac contractility, raising cardiac output and reducing blood flow and volume in non-skin areas (Crandall et al., 2008; Crandall and González-Alonso, 2010).

During HS, when the surrounding hot environment persists, the above regulation continues to function actively. A substantial volume of blood is pumped from the heart towards the peripheral blood vessels to dissipate heat through sweat, but this also results in hyperthermic dehydration of the body, reduced circulating blood volume, inadequate tissue perfusion, hypoxia and necrosis of myocardial cells (Crandall and González-Alonso, 2010; G. D; Chen et al., 2019). At the same time, the loss of body fluids disturbs electrolytes and interrupts the sodium-potassium pump, which alters the heart’s pacing rhythm, signal conduction and systolic-diastolic functional state, ultimately leading to myocardial ischemia, necrosis, arrhythmia and heart failure (Hausfater et al., 2010; Chen, et al., 2019; Tseng et al., 2019; Wang et al., 2019).

## 3 The related mechanisms of HS-induced myocardial injury

### 3.1 Dysregulation of the pro-inflammatory and anti-inflammatory balance

In HS, the systemic pro- and anti-inflammatory balance is disturbed, triggering a systemic inflammatory response syndrome (SIRS) that is thought to be characteristic (Epstein and Yanovich, 2019). The pathogenesis of heat stroke is closely similar to that of sepsis (Roberts et al., 2008). In a hot environment, the dilatation of blood vessels on the body surface due to heat dissipation leads to reduced blood flow to internal organs, especially intestinal mucosa, which causes increased intestinal epithelial permeability and bacterial translocation in the intestine, inducing leakage of intestinal endotoxins through the intestine into the circulation and triggering SIRS, ultimately leading to multi-organ dysfunction and death (Yang et al., 2007; Lambert, 2008; Leon and Helwig, 2010). The systemic inflammation associated with heat stroke plays a key role in myocardial injury. Currently, it is thought that the myocardial inflammatory response may be the primary cause of progressive systolic dysfunction (Dörge et al., 2000; Dörge et al., 2002). A large infiltration of inflammatory cells is usually found within the foci of myocardial infarction. Previous studies have shown that suppression of the inflammatory response is an important tool in the treatment of HS-induced myocardial injury (Lin et al., 2017; Lin et al., 2018; Lin et al., 2020).

During HS, the body undergoes a state of hypercytokinemia, releasing many cytokines such as tumor necrosis factor-alpha (TNF-α) and interleukin-1β (IL-1β) (Leon and Helwig, 2010; Z. T; Zhang et al., 2021). TNF-α, a key factor in the inflammatory response, plays an important role in neutrophil recruitment and the inflammatory cascade reaction (Yu et al., 2010). In addition, TNF-α induces the production of other inflammatory cytokines and also stimulates the migration and adhesion of neutrophils, leading to dysregulation of pro- and anti-inflammatory factors and inducing an inflammatory cascade reaction, which results in tissue damage (Yu et al., 2010). At the same time, the injured myocardial tissue also releases pro-inflammatory cytokines, including TNF-α and IL-6, which further exacerbate the systemic inflammatory response (Shen et al., 2019).

The TLR4/NF-κB signaling pathway has a major contribution to HS-induced inflammation. TLR4 is an essential member of the TLR family and plays a central role in the recognition and response to microbial pathogens and in maintaining the integrity of the intestinal epithelial barrier (D. Yao et al., 2019). Rats subjected to heat stress have significantly elevated levels of TLR4 (D. Chen et al., 2023). When rats are affected by heat stress, NF-κB is activated by the induced TLR4, leading to the release of pro-inflammatory factors. The production and release of pro-inflammatory factors further activates NF-κB, which induces the NLRP3 inflammasome, leading to a sustained amplification of the initial inflammatory signal, thus causing the so-called inflammatory cascade effect (Z. Huang et al., 2016; X; Zhang et al., 2017). TLR4 exhibits its highest expression in cardiac myocytes, and during HS, TLR4/NF-κB signaling controls the production of pro-inflammatory factors to induce myocardial tissue damage (X. Liu et al., 2016). Inhibition of the TLR4 signaling pathway may reduce HS-induced inflammatory responses and improve abnormal cardiac function in rats (Chen et al., 2023).

### 3.2 Endothelial cell damage and dysfunction

Cardiac ultrastructure in HS patients exhibits severe endothelial cell damage (Sohal et al., 1968). Vascular endothelial cells cover the surface of the lumen and maintain the structural integrity and microcirculatory function of the coronary microvasculature (Chang et al., 2021). It also acts as a defensive barrier against the penetration of microorganisms, immune cells and coagulation components, which reduces the risk of thrombosis (Chang et al., 2021). Activated *in vivo* crosstalk exists between vascular endothelium, inflammation and coagulation during HS (Bouchama et al., 1991; al-Mashhadani et al., 1994; Roberts et al., 2008). Endothelial cell dysfunction plays a key role in the initiation and progression of HS (W. Huang et al., 2022).

Endothelial cells possess an anti-inflammatory effect under normal physiological conditions, repelling circulating neutrophils from adhesion (Chang et al., 2021). However, when rat myocardial tissue is damaged by heat stress, endothelial cells upregulate a variety of adhesion molecules that attract pro-inflammatory cells (neutrophils and macrophages) to secrete pro-inflammatory cytokines (Harlan et al., 1991; Wihastuti et al., 2018; Chang et al., 2021). Large amounts of pro-inflammatory factors such as IL-6 and TNF-α can trigger endothelial dysfunction and microvascular damage (F. Chen et al., 2017). Damaged endothelial cells express CD40, and in the presence of CD40 interacting with CD40 ligand (CD40L), endothelial cells actively secrete von Willebrand factor (vWF), which promotes platelet adhesion to endothelial cells and contributes to thrombosis (Keuren et al., 2004; Han et al., 2018). The interaction between CD40 and CD40L also stimulates platelets and endothelial cells to activate macrophages and T cells, which further amplifies the inflammatory response (Urbich et al., 2002). Damage to the endothelium, a natural barrier against thrombosis, upregulates procoagulant factors and downregulates anticoagulant factors, thereby disturbing the dynamic balance between pro- and anti-thrombotic activities and inducing microthrombosis (Koupenova et al., 2017). Obstruction of small vessels contributes to infarction and necrosis of myocardial tissue. The damaged tissue releases plasminogen activator which induces the development of disseminated intravascular coagulation (DIC) (Sohal et al., 1968). Hearts of patients with HS show evidence of extensive visual and microscopic haemorrhage (Sohal et al., 1968).

Aspirin, a non-steroidal anti-inflammatory drug, that not only inhibits platelet aggregation but also maintains the integrity of endothelial gap junctions (Zhou et al., 2019). Animal study has shown that the treatment with aspirin significantly improves the morphological damage and related enzyme activity of chicken cardiomyocytes induced by heat stress (Wu et al., 2016).

### 3.3 Abnormal cardiomyocyte death

HS instigates multiple toxic effects on the cardiovascular system, including abnormal cardiomyocyte death (Chen et al., 2017; Chen et al., 2023). The damaged myocardial cells exhibit vacuolar changes and partial necrosis (Fan et al., 2015; Chen et al., 2019). Ferroptosis is an essential form of abnormal cardiomyocyte death caused by HS, resulting from the excessive accumulation of iron-dependent lipid reactive oxygen species (ROS) in cells, where lipid peroxidation is a key component in triggering ferroptosis (Del Re et al., 2019; Stockwell et al., 2020). HS disrupts the oxidation-antioxidant balance, as evidenced by a decrease in glutathione (GSH) and solute carrier family 7 member 11 (SLC7A11), an increase in malondialdehyde (MDA), ROS, and Fe^2+^. HS also induces shrinkage of mitochondria and an increase in the membrane density, which are key features of ferroptosis (Jiang et al., 2021; Chen et al., 2023). This suggests that ferroptosis is actively involved in HS-induced myocardial injury and causes abnormal cardiomyocyte death. Chen et al. further explored the mechanism of ferroptosis in the HS myocardial injury model (D. Chen et al., 2023). P53 expression levels were closely associated with the triggering of ferroptosis (Lei et al., 2021), and its involvement as a transcriptional repressor of SLC7A11 to ferroptosis significantly reduced the expression of SLC7A11, which in turn inhibited the activity of system X_c_^−^, a component of SLC7A11 (Koppula et al., 2018), thereby inhibiting cysteine uptake and reducing GPX4 activity leading to depletion of GSH biosynthesis (Xu et al., 2021; Zhang et al., 2022). Consequently, lipid peroxide accumulation ensued, ultimately culminating in cellular ferroptosis (Ma et al., 2022). P53, one of the molecules downstream of TLR4, is activated by the TLR4/NF-κB signaling pathway, which plays an active role in the systemic inflammatory response induced by HS (Zhu et al., 2011). In view of this, Chen et al. suggested that HS may induce ferroptosis through the TLR4/NF-κB/P53 signaling pathway (Chen et al., 2023). Inhibition of TLR4 and NF-κB under HS conditions downregulated P53 expression, upregulated SLC7A11 and GPX4 levels, improved ferroptosis-related indicators and attenuated myocardial injury, respectively (Chen et al., 2023).

Disruption of mitochondrial structure and function can lead to severe cellular damage and death (Zamzami et al., 1997; D'Orsi et al., 2017). Mitochondria plays a crucial role in maintaining intracellular calcium homeostasis (D'Orsi et al., 2017). From rat cardiomyocytes, we know that heat stress causes mitochondrial changes in cardiac myocytes including mitochondrial swelling, rupture of cristae and disruption of the surrounding membrane (Petit et al., 1998; Qian et al., 2004). Ca^2+^-ATPase on the mitochondrial membrane serves as critical factor in the regulation of calcium homeostasis. However, disruption by heat stress leads to a decrease in Ca^2+^-ATPase activity, which results in reduced mitochondrial uptake of calcium ions from the cytoplasm and intracellular calcium overload (McCormack and Denton, 1989; Walkon et al., 2022). Intracellular calcium overload further activates calcium-dependent protein kinases, which promote membrane phospholipid hydrolysis, disrupting the cytoskeleton and damaging the integrity of the nucleus, causing severe damage (Vassalle and Lin, 2004). HS directly induces the opening of mitochondrial mPTP, a pivotal event in triggering the cell death pathway (Halestrap, 2009; Bauer and Murphy, 2020). mPTP opening results in a series of cytological effects that lead to the release of cytochrome c, activation of caspase family proteases and apoptosis of cardiomyocytes (H. Yao et al., 2022). The mechanism by which HS induces mPTP opening is not yet clear, and the Fas pathway is an important signaling pathway to consider. It induces caspase-8 activation, which subsequently directly activates caspase-3 and leads to the opening of mPTP (Nakamura et al., 2000). However, whether the Fas pathway is involved in HS-induced mPTP opening remains to be explored.

### 3.4 Metabolic abnormalities

The link between metabolic dysregulation and cardiotoxicity has been well established (Russo et al., 2021). Mitochondrial damage caused by HS not only results in abnormal death of cardiomyocytes but also leads to disturbances in energy metabolism (Azevedo et al., 2013). Energy abnormalities in the heart are associated with the development of many heart diseases (X. Wang et al., 2023). Heat stress disrupts the integrity of the mitochondria, which is the basis for normal mitochondrial function, resulting in a suppression of energy production from the oxidative metabolism of cardiomyocytes (Patra and Hay, 2014; Laitano et al., 2020; Deng et al., 2022). However, in response to the high temperatures of the external environment, the heart requires a greater supply of energy to enhance cardiac function, which leads to a significant decrease in the ATP content of the cardiomyocytes and eventual death due to energy deficiency (Qian et al., 2004).

Glucose and fatty acids are essential substrates for oxidative phosphorylation. Glucose and lipid metabolism plays an important role in cardiac myocytes by providing energy and maintaining cellular function (H. Tian et al., 2023). However, studies in murine models of EHS have revealed that HS alters cardiomyocyte metabolic pathways, disrupts the glycolytic and oxidative phosphorylation pathways by upregulating glycolysis-related enzymes, thereby enhancing lactate production to impair cardiomyocyte function (Laitano et al., 2020). The perturbation of glucose and lipid metabolism by HS may be related to the inhibition of the AMPK signaling pathway (Rodríguez et al., 2021). AMPK increases ATP production in cardiomyocytes through stimulation of glucose metabolism and fatty acid oxidation. AMPK phosphorylation at Thr^172^ induces acyl CoA-carboxylase (ACC) phosphorylation to inhibit the conversion of acetyl-CoA to malonyl-CoA during fatty acids (FAs) synthesis (Carling et al., 2008). Beyond the inhibition of lipid anabolism, p-AMPK also promotes FAs uptake by inducing the activity of the FAs transporter CD36, enhancing β-oxidation (Habets et al., 2009). Glucose metabolism is also regulated by AMPK. p-AMPK increases glucose transporter 4 (GLUT4), which promotes glucose uptake and thus provides a source of energy (D. Zheng et al., 2001). Under HS conditions, phosphorylation of AMPK is inhibited, leading to dysregulation of glucolipid metabolism and disruption of energy metabolism (Roths et al., 2023). This ultimately leads to cell death and impaired cardiac function. Therefore, targeting glucose and lipid metabolism may be an effective way to counteract HS-induced myocardial injury.

### 3.5 Heat shock protein dysregulation

Cells from a murine model of myocardial tissue turn on their intrinsic defense mechanisms in the face of heat injury, with a dramatic increase in heat shock protein (HSP) expression being a key part of the heat shock response (Tang et al., 2013; Tang et al., 2016). It can interlock with apoptosis, inflammation and autophagy to regulate cellular homeostasis and prevent tissue damage (Hsu et al., 2013; Shen et al., 2019). It was mentioned earlier that patients with HS can develop severe vascular endothelial cell damage. After heat exposure, strong positive signals for HSP90 and HSP70 are detected in rat cardiac microvascular endothelial cells, helping the vascular endothelium to resist heat injury (X. Zhang et al., 2020). An increase in HSP90 activates the PI3K/Akt signaling pathway. Phosphorylated Akt negatively regulates the expression of pro-apoptotic proteins and contributes to cell survival (Zhang et al., 2020). It is known from rat-related experiments that HSP levels vary with the duration of heat stress. In the early stages of HS, HSP rises sharply, and as time progresses, HSP is heavily depleted, resulting in abnormally low HSP levels in the later stages (H. B. Chen et al., 2015; Lin et al., 2020). When HSP90 is crushed, the interaction of HSP90 with Akt is reduced, weakening the protective effect. This results in the vascular endothelium exhibiting a more sensitive state to heat stress and more severe damage (Zhang et al., 2020).

## 4 The treatment strategy for HS

The prognosis of patients with heat stroke is directly related to the degree and duration of the increase in core temperature (Hadad et al., 2004). Therefore, whole-body cooling is the current treatment of choice for HS. Following the onset of HS, hypotension and altered cardiac protein profiles are demonstrated, which can be reversed by whole-body cooling (Ko et al., 2020). Temperature reduction is achieved mainly by conduction, evaporation and convection (Hadad et al., 2004). In addition, symptomatic support therapy is an integral part of the treatment. When hypotension occurs in patients, aggressive fluid resuscitation and vasoactive medication should be administered with the avoidance of alpha-adrenergic drugs as they exacerbate peripheral vasoconstriction and inhibit core body temperature reduction (Atha, 2013; Asmara, 2020). Excessive inflammation and coagulation disorders are important pathogenic mechanisms of HS, therefore anti-inflammatory and anticoagulant therapies are also available as treatment options (Y. F. Tian et al., 2013; Kobayashi et al., 2018). For patients who progress to multi-organ dysfunction despite hypothermia treatment, continuous blood purification and plasma exchange are often selected, aiming not only to alleviate the body’s catabolic state but also to eliminate inflammatory mediators from the bloodstream to facilitate the recovery of HS patients (Wakino et al., 2005; K. J; Chen et al., 2014). Given the danger and intractability of HS, prevention strategies are far more beneficial than any present treatment strategies. People at risk of heat exposure should be thermally acclimatized in advance, with consumption of sufficient fluids and adequate nutrition (Asmara, 2020).

## 5 Discussion

HS involves a complex biochemical cascade of reactions and is caused by a combination of factors. The cardiovascular system is considered to be the first system affected by HS. Circulatory shock occurs in approximately 20%–65% of patients, and an even higher 85% of patients will develop ECG abnormalities (Austin and Berry, 1956; Asmara, 2020). However, a dearth of clinical directives exists regarding the efficacious management of cardiovascular ailments amidst elevated temperatures. A precise comprehension of the fundamental mechanisms whereby heightened temperatures inflict harm upon myocardial tissue is imperative to judiciously formulate preventative and therapeutic strategies. This review summarizes the possible pathogenesis of HS-induced myocardial injury, which can help provide new targets for the treatment of HS.

The predominant body of research scrutinizing myocardial impairment due to hyperthermia predominantly comprises animal studies, with a paucity of involvement from clinical cohorts. The acquisition of clinical data assumes heightened significance. A comprehensive database analysis encompassing 27 countries spanning the years 1979–2019 revealed a 7% escalation in mortality among patients with ischemic heart disease during episodes of soaring temperatures (Alahmad et al., 2023). Moreover, a meta-analysis delineated a 2.8% augmentation in the risk of developing coronary heart disease for each 1°C ascent in temperature (J. Liu et al., 2022). Endothelial cell damage within cardiac vasculature due to pyrexia precipitates thrombosis, culminating in acute coronary incidents. Clinical investigations have documented a substantial surge in hospitalizations linked to coronary artery disease following exposure to elevated temperatures (Fuhrmann et al., 2016). Long-term monitoring of hyperthermia-stricken patients corroborates the critical role of intact myocardial tissue, with a meager 1-year survival rate of merely 24% observed in cases with markedly elevated troponin levels (Marchand and Gin, 2022). This underscores the profound impact of myocardial impairment on the prognosis of hyperthermia-afflicted individuals. Consequently, the primary focus of research should pivot towards averting myocardial damage induced by HS.

The incomplete comprehension of HS pathogenesis, coupled with the absence of evidence-based medical guidance for clinical interventions, has resulted in the inadequacies of current treatment modalities. Predominantly, supportive therapies such as whole-body cooling and fluid resuscitation constitute the primary approach. Regrettably, a lack of standardized endpoint objectives for whole-body cooling persists to date. Furthermore, despite numerous animal studies affirming the favorable efficacy of anti-inflammatory and anti-endotoxic agents for HS, their translation into clinical success remains limited. Aspirin, despite demonstrating effectiveness against heat-induced injury in avian cardiac tissues, fails to manifest any clinical benefit and may potentially exacerbate coagulation disorders and hepatic dysfunction (Tek and Olshaker, 1992). Individuals with cardiovascular ailments not only contend with the vulnerability of their cardiac systems in the face of HS but also grapple with an elevated risk due to commonly prescribed cardiac medications. β-blockers impede the capacity to augment cardiac output in response to HS, while diuretics exacerbate hypovolemia and elevate the risk of electrolyte imbalances (Marchand and Gin, 2022). This begs the question of which medications can be initiated or stopped during extreme heat conditions.

In cases where the heart receives heat damage, significant changes in cardiac metabolism occur. These changes are not only passive bystanders, but are actual participants in causing heat stress damage to the myocardium. Targeting myocardial metabolism could be the tool for our effective treatment. Interventions in cardiac metabolic processes have been successfully used to reduce infarct size in animal models of myocardial ischaemia-reperfusion injury (Valls-Lacalle et al., 2018; Zuurbier et al., 2020). However, suitable drug targets for conversion in patients with acute myocardial infarction are still awaited. Cardiometabolic therapies are challenging, but fortunately, recent methodological advances in detecting metabolic changes within the heart will make our efforts more achievable.

In conclusion, HS-induced myocardial injury arises from a combination of excessive inflammation, endothelial cell damage, abnormal cardiac metabolism, and heat shock protein dysregulation. In the treatment, in addition to systemic supportive therapy it should also focus on precise targeting of myocardial tissue. Only with a deeper and clearer understanding of the mechanisms underlying the development of HS will there be an opportunity to establish more effective treatment.

## 6 Future perspective

Given the increasing mortality rate associated with HS, extensive research has been conducted to explore this condition. A meticulous examination of the literature has revealed potential molecular targets for HS treatment, encompassing TLR4, P53, AMPK, and HSP. Additionally, the Fas signaling pathway presents a novel avenue for HS management. However, the majority of these investigations have been confined to the realm of animal studies, and the therapeutic strategies delineated await clinical validation. Consequently, we should focus more on clinical trials to find relevant drug targets that can serve clinical HS patients. Furthermore, the absence of targeted therapy for HS-induced myocardial injury underscores the need for advancements in this area. Fortunately, the rapid development of modern bioinformatics technologies offers us valuable tools to deepen our understanding of the pathogenesis of HS-induced myocardial injury and implement precise treatments.

## Abbreviations

HS, Heat stroke; CHS, Classic heat stroke; EHS, Exertional heat stroke; SIRS, Systemic inflammatory response syndrome; TNF-α, Tumor necrosis factor-alpha; IL-1β, Interleukin-1β; NLRP3, NOD-like receptor family pyrin domain containing3; vWF, von Willebrand factor; DIC, Disseminated intravascular coagulation; ROS, Reactive oxygen species; GSH, Glutathione; SLC7A11, Solute carrier family 7 member 11; MDA, Malondialdehyde; ACC, Acyl CoA-carboxylase; FAs, Fatty acids; GLUT4, Glucose transporter 4; HSP, Heat shock protein.
